# The costs of suboptimal breastfeeding in Ontario, Canada, and potential healthcare resource impacts from improving rates: a pediatric health system costing analysis

**DOI:** 10.1186/s13006-025-00702-y

**Published:** 2025-02-22

**Authors:** Nnachebe Michael Onah, Sandra Hoy, Kathleen Slofstra

**Affiliations:** 1 Community Healthcaring Kitchener Waterloo, Kitchener, ON N2G 2A2 Canada; 2 School of Social Work, Laurentian University, Sudbury, ON Canada

**Keywords:** Suboptimal breastfeeding rates, Canadian healthcare cost analysis

## Abstract

**Background:**

Human milk from the breast is the healthiest option for infants. Other sources of nutrition pose some risk to child, maternal, and environmental health. There are significant costs to suboptimal rates of breastfeeding for children, families and society. Over 92% of mothers in Ontario, Canada initiate breastfeeding, yet exclusivity and duration rates decline over time. This study estimates potential pediatric healthcare cost savings from increased exclusive breastfeeding.

**Methods:**

We conducted a cost-effectiveness analysis to compare healthcare savings from enhanced breastfeeding rates against current practices by estimating pediatric healthcare costs associated with suboptimal breastfeeding and potential savings from improved rates. Savings are calculated from reduced incidence of childhood illnesses associated with breastfeeding, including lower respiratory tract infections (LRTI), gastrointestinal infections (GII), acute otitis media (AOM), acute lymphoblastic leukemia (ALL), necrotizing enterocolitis (NEC), childhood obesity, and asthma. Cost data were drawn from Canadian healthcare sources, supplemented with data from the UK and other international studies. We used initiation and exclusive breastfeeding rates at hospital discharge and six months postpartum.

The study assumes that the incidence of preventable conditions like LRTI, GII, and AOM is directly related to breastfeeding rates at these time points. A six-month threshold for exclusive breastfeeding, recommended by the World Health Organization, was selected for analysis. Partial breastfeeding rates were not separately modeled due to data limitations.

**Results:**

Improving exclusive breastfeeding (EBF) rates at six months to match rates at hospital discharged and initiation rates could result in 47,114–91,457 fewer cases of LRTI, GII, and AOM, prevent 3,685–7,096 hospitalizations, and reduce 22,043–47,621 outpatient visits. Increased EBF rates could prevent cases of NEC (37–67), ALL (3–6), childhood obesity (1,199–2,661), and asthma (970–2,111). Suboptimal breastfeeding at 6 months for infants born in Ontario in 2019 cost the healthcare system US $72.2 million annually for treating four childhood illnesses and US $61.0 million for long-term conditions (ALL, obesity, and asthma). Increasing breastfeeding rates could save US $32–63 million in annual treatment costs and US $23.6–51.6 million in long-term healthcare costs.

**Conclusions:**

Suboptimal breastfeeding rates impose a burden on the health of families and Ontario’s healthcare system. Supporting breastfeeding through evidence-based interventions could reduce this burden through lowering pediatric healthcare demands.

**Supplementary Information:**

The online version contains supplementary material available at 10.1186/s13006-025-00702-y.

## Background

Human milk from the breast is the healthiest option for human infants. All other sources of nutrition pose some risk to child, maternal, and environmental health [1]. Interventions that facilitate breastfeeding and support families are cost-effective public health investments [2]. For children to obtain optimal growth and development, the World Health Organization and Health Canada recommend initiation of breastfeeding within the first hour after birth; exclusive breastfeeding for the first six months; and continued breastfeeding for two years or more, with complementary feeding starting at six months [3].

Canada’s healthcare system is publicly funded, providing universal access to medically necessary services without direct costs to patients. Each province administers its own system under federal guidelines. In Ontario, the Ontario Health Insurance Plan (OHIP) covers essential healthcare services, including hospital care and physician visits. The public system covers the pediatric assessment, diagnosis and some of the treatments for the illnesses in this study. Some services like dental care and prescription medications may require private insurance or out-of-pocket payments. In Ontario, the most populous province in Canada, most families want to feed infants human milk as breastfeeding initiation rates are an impressive 92.1% [4]. However, exclusivity rates fall to 64.9% at hospital discharge, to 36.3% at 6 months after birth [4, 5]. Limited access to breast milk banks exist and human milk feeding (mother’s own milk and/or donor human milk) rates for infants in the neonatal intensive care unit (NICU), are particularly low, with one report finding that only 40.6% of preterm (< 37 weeks) newborns are receiving any human milk at NICU discharge to home [6].

Notably, breastfeeding in Canada is influenced by social determinants of health. Children of white, educated, older, middle-class women are most likely to be fed human milk and for longer periods of time [7]. Breastfeeding advocates and researchers conclude that to increase duration rates, we must surround families with relevant, evidence-based, breastfeeding friendly health and social supports that address the diversity of challenges to establishing the breastfeeding relationship [2].

A global review of the health and societal benefits concluded that increasing breastfeeding to a near universal level could prevent 595,379 childhood deaths from diarrhoea and pneumonia each year, reduce 974,956 cases of childhood obesity, and prevent 98,243 deaths from breast and ovarian cancers as well as type II diabetes each year [8]. These avoidable deaths and diseases involve costs of premature death, indirect costs and health system treatment costs of US$1.1 billion annually. Including losses associated with the cognitive impact of not breastfeeding, the total global economic losses estimated to be US$341.3 billion, or 0.70% of global gross national income [8]. Country level cost analysis studies reporting the costs of suboptimal breastfeeding rates on healthcare systems have been conducted in US [9, 10], UK [11], Mexico [12, 13], South East Asia [14], Spain [15, 16], and within a number of other healthcare systems internationally [8]. Each study has demonstrated economic savings the healthcare system would see in terms of reduced costs associated with reduced disease burden and death as breastfeeding rates increase.

The costing study we located in Canada is a Newfoundland data linkage study that found exclusively formula fed infants had higher average spending associated with hospital admissions, family doctor and specialist visits, and both mixed feeding and exclusively formula feeding was predictive of higher total healthcare service use [17].

In the present study, we sought to estimate the pediatric illness costs of suboptimal breastfeeding rates to the Ontario healthcare system and model the healthcare cost savings if breastfeeding rates in Ontario, Canada were increased. The project involved updating the systematic reviews by Renfrew et al. [11] and replicating their efforts in a Canadian context. To our knowledge, no such costing data has been reported for Canada. A Canadian study is an important contribution to the international, national, and provincial discussions related to investments in breastfeeding supports as preventative healthcare policy and practice in high-income countries.

## Methods

A health system costing analysis from a provider perspective was used to model potential cost savings to the Ontario health system if breasting initiation or hospital discharge rates are sustained at six months. The costing analysis estimates potential annual cost savings that the Ontario health system could achieve from treating annual breastfeeding-attributable pediatric diseases (LRTI, AOM, GII, and NEC). For ALL, we estimated the childhood (14 years) cost of treating attributable cases. For childhood obesity and asthma, we estimated excess healthcare costs attributable to disease cases per annum over 14 years. Childhood costs were determined based on disease commencement before age 5 and costs incurred before children turned 19 years old. Costs were obtained from published estimates from hospital records (LRTI, AOM, GII, and NEC) and from the literature (ALL, obesity, and asthma). Specifically, we estimated the treatment and management cost savings for the health system that could result from higher breastfeeding rates in Ontario, Canada. We chose to build on the work of Renfrew et al. [11] as we aimed to avoid overestimation of costs and chose to err on the side of conservative assumptions when making methodological decisions. Thus, the true scale of the impact of infant feeding on the Ontario healthcare system is likely much greater.

Renfrew’s study followed a multi-step approach that first identified the association between breastfeeding and the reduction in incidence rates of several childhood illnesses. The study then estimated the healthcare costs associated with treating these conditions using data from the UK National Health Service. They used breastfeeding prevalence rates and compared current breastfeeding practices with hypothetical increased rates of breastfeeding exclusivity and duration. Cost savings were calculated by multiplying the number of prevented cases by the average healthcare costs associated with treating each condition. In our study, we adapted the Renfrew methodology to the Ontario context by building on their literature review and using Ontario-specific breastfeeding initiation, exclusivity, and duration rates. We similarly modeled the incidence of preventable pediatric illnesses such as LRTI, GII, and AOM. Additionally, we applied Ontario healthcare cost data to estimate the potential cost savings, while following the same framework used by Renfrew et al. to link increased breastfeeding rates with the prevention of childhood illnesses.

The costing perspective was that of the Ontario health system. We utilised 2017–2018 [4, 5] breastfeeding rates for modelling current health system cost burden and compared costs with scenarios of improved breastfeeding rates. We used rates from data collected between 2017 and 2018 since we could not find 2019 rates for Ontario. While this rate might vary from 2019, this was the best available estimate during modelling. These increases were modelled based on disease-specific scenarios; using 2017-18 breastfeeding initiation rates at birth, at hospital discharge after birth, and at six months [4, 5]. To achieve this, our methodology included literature reviews to identify input parameters and cost estimates. Our modelling focused on the 2019 live births.

### Disease selection and literature searches

The process of selecting seven priority illnesses was based on an extensive search of the literature. Using our university library access to common databases, we reviewed and updated the systematic reviews conducted by Renfrew et al. [11] to identify high-quality studies and parameters to include in the model where Canadian data was missing. In addition, since we modelled three illnesses that were not included in Renfrew et al. study (acute lymphoblastic leukemia, childhood obesity, and asthma), we applied the same approach in literature reviews and data extraction. Refer to Supplemental File 1 for details of the search strategy. The results of the reviews can be found in Supplemental Files 2–4. Review A involved updating the systemic search for existing systematic reviews of infant feeding and health and cognitive outcomes in high income countries by Renfrew et al. [11] (Supplemental File 2). Review B was a systematic search and identification of Canadian studies (Renfrew’s Review B was a focus on UK studies) examining health outcomes related to infant feeding (Supplemental File 3). Review B offered a picture of the available Canadian data on breastfeeding outcomes. Review C was a review of economic impact (cost of illness) studies related to infant feeding from comparable industrialized, high-income countries and informed our overall approach to the study (Supplemental File 4).

For Review A, we developed a screening tool with exclusion and inclusion criteria and another researcher applied the screen to a random 10% to check reliability of the screen. The Review A screen contained the following inclusion criteria: review studies must be published between 2012 and 2023, have a design described by the author(s) as a “systematic review” or “review,” include reviews of studies with participants from high income countries or middle income countries, review studies where some or all participants breastfed and/or fed with breast milk (includes hand expression and pumped breast milk fed through means other than the breast (cup, bottle, lactation device etc.) and a focus on measuring the relationship between breastfeeding and some form of pediatric health and cognitive outcomes. The screen for Review B required that studies have designs described as “controlled,” “experimental” or “epidemiological” (observational or analytic), “descriptive single-group cross‐sectional” or “longitudinal” and include some analysis of the relationship between breastfeeding and pediatric health and cognitive outcomes and include participants from Canada. For Review C, the articles must have been published between 2012 and 2023, include a cost of illness associated with not breastfeeding analysis, include participants from developed/high income countries or transitioning/emerging economies/middle income countries, include an analysis of participants breastfeeding and/or feeding with breast milk (includes hand expression and pumped breast milk fed through means other than the breast (cup, bottle, lactation device etc.) and an analysis of the healthcare resource implications of treating conditions that could have been prevented by breastfeeding or resource implications of other public services of meeting needs that could have been prevented by breastfeeding.

Using the approach outlined by Renfrew et al. [11] we had a series of meetings with the research team to discuss the state of the evidence from Reviews A and B. We also consulted with two Canadian researchers with expertise in specific diseases relationships to breastfeeding in our decision-making process. We chose childhood illnesses with the most robust evidence of association with breastfeeding, where review studies were available to predict the effect size with confidence, and where it was possible to conduct an economic analysis that was relevant to the Ontario healthcare system.

With the results from our analysis of reviews A and B, we chose to model the cost burden and potential cost savings from healthcare expenditures incurred by Ontario’s health system in treating seven childhood illnesses: lower respiratory tract infection (LRTI), gastrointestinal infection (GII), acute otitis media (AOM), necrotizing enterocolitis (NEC), child acute lymphoblastic leukemia (ALL), child obesity, and asthma. A number of diseases with relationships to breastfeeding were excluded from our analysis. We excluded diseases where there is currently a lack of a strong evidence of association, diseases where there are complicated relationships with feeding modality, diseases with limited key Canadian data, and diseases where there were challenges with modelling the disease impact on the Ontario healthcare system. Our inclusion and exclusion decisions were also guided by a concern for containing the scope of the study. Once the shortlist of outcomes was determined, further online database and literature searches were carried out to identify the following model parameters: breastfeeding rates (current practice); incidence of outcomes; incidence of care episodes (outpatient consultations, and hospitalisation) specific to the selected conditions; unit costs of treatment or management for each condition or unit-costs of care episodes.

### Cost modelling framework and assumptions

**Fig. 1:**
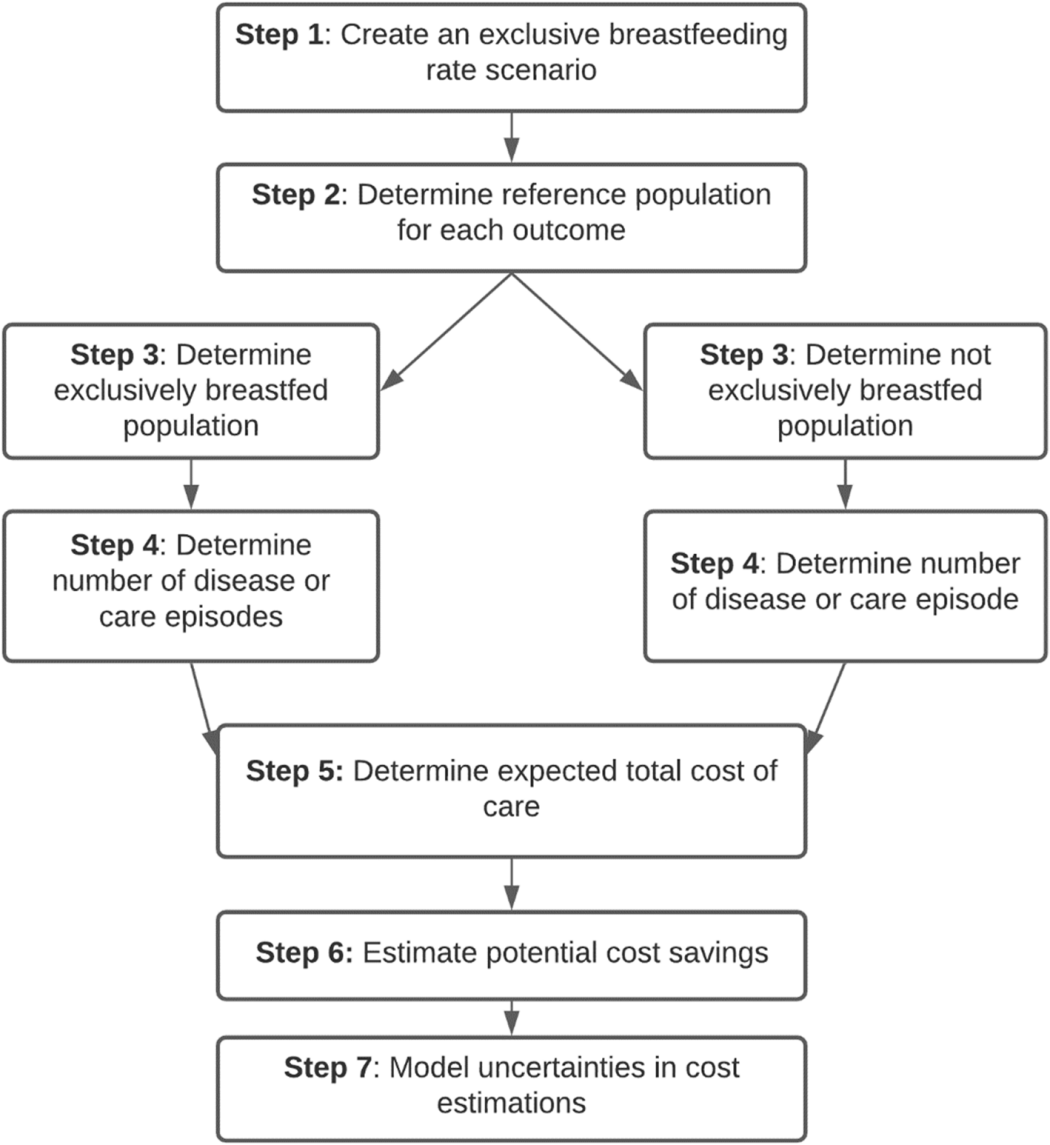
Process of economic modelling. Adapted from Renfrew et al. [11]

We utilised a 7-step framework for modelling the identified outcomes. This framework builds on common methods utilised in previous studies [9, 11] and is illustrated in Fig. 1.

A full list of modeling assumptions per disease can be found in Table 1. For step one, we modelled breastfeeding scenarios that are disease-specific. For AOM, GII, LRTI, child obesity, and asthma, we used three exclusive breastfeeding rate scenarios: (1) current rate (36.3%) at six months; (2) increasing current rates at six months to rates at hospital discharge (64.9%); (3) increasing current rate at six months to initiation rates at birth (92.1%). For NEC, we modelled three different breastfeeding scenarios, (1) current rate of any breastfeeding at NICU discharge (32.9%); (2) increasing rate at discharge to rate of any breastfeeding at 6 months (64.6%); (3) increasing rate at NICU discharge (32.9%) to initiation rate (92.1%). These Ontario breastfeeding rates were chosen based on the accepted practice in other costs analysis studies, and the availability and reliability of infant feeding data from sources in Ontario. The initiation and 6 month exclusivity data is reported in a Public Health Agency of Canada report [4] and is from Canadian Community Health Survey data (2017–2018). The exclusive feeding at hospital discharge rate is reported by Baby-Friendly Initiative Ontario (BFION) [5] and is from BORN (Better Outcomes Registry & Network) Ontario data collected in participating Ontario hospitals. For childhood ALL, we modelled three breastfeeding scenarios; (1) current rate of any breastfeeding at 6 months (64.6%); (2) increasing rates of any breastfeeding at 6 months (64.6%) to rates at hospital discharge for full-term births (73.8%); (3) increasing of any breastfeeding at 6 months (64.6%) to initiation rates (92.1%) (see Table 1). For step two, the reference population was the number of live births. We adjusted the number of live births with estimates of infant and neonatal mortality rates obtained from Statistics Canada [18, 19]. For NEC, we used an 8% rate of preterm births in Canada of which 14.30% are considered early preterm (i.e. <32 weeks gestational age) [20]. In step three, the reference population was divided into two groups based on their breastfeeding status. To determine the number of disease cases and care episodes for each breastfeeding scenario, we utilised two approaches. For LRTI, AOM, GII, and NEC, we relied on evidence from literature of the incidence of illnesses based on breastfeeding status (exclusively breastfed [EBF] and artificial or formula fed [FF]) (see Supplemental File 1). For childhood ALL, child obesity, and asthma, we relied on a differential incidence equation first described by Bartick and Reinhold (2010) and again utilized by Renfrew et al. [9, 11]. The equation used is $\:x=s/\left(br+1-b\right)$, where $\:x$ is the diseases incidence in non-breastfeeding group, $\:s$ is the overall incidence of identified disease, $\:b$ is the current breastfeeding rate, $\:r$ is the odds ratio in favour of breastfeeding, and $\:xr$ is the incidence of disease in breastfeeding group. We used the formula when the odds ratio approximates the risk ratios. The difference between number of illness cases based on breastfeeding scenarios (Scenario A – Scenario B; Scenario A – Scenario C) were considered as numbers of cases prevented. Applying the estimates above in step five, we determined the number of children with outcomes of interest and obtained the incidence of care episodes where needed and multiplying this with the unit cost of care per episode disaggregated by inpatient and outpatient costs where available.

**Table 1:** Key data sources and assumptions

Parameter	Description			Source
Number of live births	140,541 live births in Ontario (2019)			Statistics Canada [18]
Infant and neonatal mortality rate	4.6 infant and 3.6 neonatal deaths per 1000 live births			Statistics Canada [19]
Breastfeeding rates	36.3% (exclusive rate at six months); 64.9% (exclusive rate at hospital discharge after delivery); 92.1% (breastfeeding initiation rate); 73.8% (any breastfeeding rate at discharge) 64.6% (any breastfeeding rate at 6 months);32.9% any breastfeeding at NICU discharge			PHAC, 2022 [4]; BFI Annual report, 2019 [5];
**Lower respiratory tract infection**				
Incidence of LRTI	37% among FF and 25% among EBF			Quesada et al. 2020 [21]
Incidence of care	23% cases visit outpatient care; 6% have inpatient stays			Renfrew et al. 2012 [11]; CIHI [22]
Duration of care	3 days in inpatient care per episode; 1.9 days outpatient visits per episode			OCCI [23]; CIHI [22]
Cost of care	US $911 (US $222- US $8,112)* per inpatient stay; US $166 (US $99 – US $11,660)* per LRTI case in outpatient care			OCCI [23]
**Gastrointestinal infection**				
Incidence of GI	31% among FF and 14% among EBF			Quesada et al. 2020 [21]
Incidence of care	22% cases visit outpatient care; 44% have inpatient stays			Sargeant et al. 2008 [24]; Caudle et al. 2009 [25]
Duration of care	3.6 days in inpatient care per episode; 1.9 days of outpatient visits per episode			OCCI [23]; CIHI [22]
Annual cost of care	US $1,055 (US $810 – US $9088)* per inpatient stay; US $166 (US $144 – US $1,338)* per GI case in outpatient care			OCCI [23]
**Acute otitis media**				
Incidence of AOM	37% among FF and 25% among EBF			Quesada et al. 2020 [21]
Incidence of care	94% cases visited a health professional			Dubé et al. 2011 [26]
Duration of episode	2.2 AOM episodes per annum; 5.9 days per AOM episode			Dubé et al. 2011 [27]
Annual cost of care	US $97.71 (US $ 87 – US $1,919)* per AOM case in outpatient care			OCCI [23]
**Necrotising enterocolitis**				
Number of preterm babies	About 1.16% of live births are early preterm (< 32 weeks gestation age) in Canada			CIHI 2009 [22]
Breastfeeding rates	32.9% of premature newborns are EBF and 40.6% are mixed fed at NICU discharge			CNN [6]
Incidence of NEC	7% among AF and 1% among exclusively breastmilk fed (EBF)neonates			Quesada et al. 2020 [21]
Incidence of care	We assume all newborns with NEC receives care			Analyst’s assumption
Duration of care	32.9 days in inpatient care days per admission			OCCI [23]
Distribution of treatment	31% of cases would need surgical treatment while 69% would need medical treatment only			Rees et al. 2010 [28]
Annual cost of care	US $60, 326 (US $42,035 – US $87,658)* per NEC case treated in NICU			OCCI [23]
**Childhood acute lymphoblastic leukemia**				
Incidence of acute lymphoblastic leukemia	7.0 cases per 100,000 population of 0–4-year-olds in Ontario			Statistics Canada [29]
Probability of infection	1.70% probability of developing acute lymphoblastic leukemia for < 5 years olds in Canada			CCSAC [26]
Probability of death	0.9% probability of dying from acute lymphoblastic leukemia in Canada			CCSAC [26]
Survival rate	94% predicted 5 year observed survival proportion			CCSAC [26]
Breastfeeding and acute lymphoblastic leukemia risk	0.84 (0.75–0.94) odds ratio for any breastfeeding for ≥ 6 months vs. <6 months; 0.91 (0.8–0.94 CI) odds ratio for ever breastfed vs. never breastfed			Amitay et al. 2015 [30]
Healthcare seeking	About 92% of cases sought care at pre-diagnosis and initial phase, 85% will spend and seek care time at continuing care phase, and 18% would enter terminal phase and accumulate healthcare cost in Ontario, Canada.			McBride et al. (2020) [31]
Mean (14 year)cost of care	US $3,919.70 pre-diagnosis care, US $125,034 initial/1-year post diagnosis care, US $17,141.6 continuing care, and US $345,759.1 terminal care phase in Canada.			McBirde et al. 2020 [32]
Child obesity				
Prevalence rate	13.1% of children aged 1–19 are obese in Canada			Rao et al., 2016 [33]
Breastfeeding and child obesity risk	0.66 (0.50–0.88 CI) odds ratio in favour of EBF at 6 months vs. FF			Ma et al. 2020 [34]
Excess healthcare use	Childhood obesity results in an increase in annual per capita healthcare cost by US $305.72 (US $241.39 – US $374.04)			Ling et al. 2022 [35]
Pediatric asthma				
Incidence rate	24.8 per 1000 person-years among zero to 10 year olds in Ontario Canada			Radhakrishnan et al. 2021 [36]
Breastfeeding and child asthma risk	0.70 (0.53–0.92 CI) odds ratio in favor of EBF vs. not EBF			Xue et al. 2021 [37]
Age at onset	In Canada, 39.9%, 42.9%, and 17.2% of children had the first asthma or wheezing diagnosis at between 0–3 years, 4–7 years, and 8–12 years of age, respectively			Chen et al. 2021 [31]
Annual cost	Pediatric asthma results in a US$828.86 (US $761.67 – US $1,304.75) increase in annual healthcare expenditure			Ungar et al., 2001 [38]

*FF*  Formula fed, *EBF* Exclusively breastfed, *CIHI* Canada Institute of Health Information, *OCCI* Ontario Case Costing Initiative, *CCS* Canadian Cancer Society, *US$* United States dollar, *NICU* Neonatal Intensive Care Unit, *CNN* Canadian Neonatal Network, *CIHI* Canada Institute of Health Information, *PHAC* Public Health Agency of Canada, *CCO* Cancer Care Ontario

*min and max cost estimates from OCCI

We estimated annual healthcare costs for AOM, LRTI, GII, and NEC. For ALL, childhood obesity and asthma, we estimated excess healthcare costs attributable to illness over their childhood years broadly defined as disease onset as age five and cost incurred over 14 years before adulthood. For AOM, LRTI, GII, and NEC, we obtained annual medical and healthcare management costs attributable to illness. For childhood ALL, we used childhood (14-years) cost to estimate healthcare costs from McBride et al. [32]. This was disaggregated based on healthcare seeking proportions (i.e. proportion of cases who utilised care at different illness phases [pre-diagnosis, initial/first-year, continuing care, and terminal care phases]). For child obesity, we estimated the excess per annum healthcare expenditure attributable to child obesity over 14 years from Ling et al. [35]. For childhood asthma, we estimated the annual attributable healthcare costs from Ungar and Coyte [38] and estimated childhood (14 years) healthcare costs using age distribution at disease onset from Chen et al. [31]. In step six, annual treatment or childhood (14 years) healthcare cost per each modelled breastfeeding scenario were subtracted from the current rate to obtain cost savings. Since input parameters were obtained from multiple sources with different methodologies including sample design, sample size, and definition of breastfeeding, to examine the uncertainties in modelled costs, we used deterministic sensitivity analyses to examine the impact of varying the odds ratios and unit costs used in the models. To do this, we utilised the confidence intervals in the odds ratio in favour of breastfeeding for child ALL, obesity, and asthma. In addition, for AOM, LRTI, GII, NEC, child obesity, and asthma, we used low and high bound estimates of unit treatment costs.

### Costing approach and perspective

The cost model for estimating the healthcare costs of select pediatric illnesses attributable to suboptimal breastfeeding can be summarised using the equation below:

1 $$\:C\left(\beta X_{it}\right)={\beta_0\:}+{\sum\:}_i\beta_jX_{ijt}$$

where $\:{X}_{it}$ is a vector of explanatory variables (predictors of cost) for patient $\:i$ in period $\:t$ whose $\:{j}^{th}$ element is $\:{X}_{ijt}\:$and $\:\beta\:\:$is a vector of regression coefficients whose $\:{j}^{th}$ element is $\:{\beta\:}_{j}$. $\:\beta\:$ is unique to particular treatment algorithm and $\:t\:$represents a specific time period, so that the basic unit of observation is the patient-time.

The costing approach utilised a health system perspective focussing on specific cost centres. Unitary costs for inpatient, outpatient, and medication care were estimated where available else, we relied on treatment cost for illnesses. We relied on the reviews for obtaining estimates of key parameters that are drivers of healthcare costs. These estimates covered incidence of inpatient and outpatient care visits per disease episode, and average length of inpatient stays and outpatient visits. Table 1 presents a summary of key parameters and data sources that inform this costing study. We relied on the Ontario Case Costing Initiative (OCCI) for inpatient and outpatient costs for LRTI, GII, AOM, and NEC, and on literature for treatment costs for childhood (ALL), child obesity, and asthma [23]. For childhood ALL, child obesity, and asthma we discounted future treatment costs to 2020 costs using a 3% discount rate. Cost parameters obtained from the literature review were inflated/deflated to 2020 Canadian dollars using the Bank of Canada inflation calculator [39] and then converted to the United States dollar using exchange rate estimates from the World Bank [40].

Finally, to explore how sensitive our estimates are based on chosen input parameters and since all relationships between breastfeeding and illness outcomes are associations, we performed deterministic sensitivity analysis by varying some of the disease and cost parameters. We varied treatment costs using minimum and maximum costs from the OCCI [23], and disease odds ratios using confidence intervals. Analysis was performed in Microsoft Excel. We used Excel to produce estimates based on the aforementioned 7-step economic modelling approach.

## Results

The following results are based on an extensive review of existing literature, healthcare databases, and cost analyses, which aimed to assess the impact of suboptimal breastfeeding rates on childhood diseases and healthcare costs in Ontario. The findings presented summarize key insights from this review and are supported by detailed data found in the supplementary files.

### Potential cost savings

#### Lower respiratory tract infections

Pediatric LRTIs (e.g. bronchitis, pneumonia, bronchiolitis and respiratory syncytial virus [RSV] and others often caused by influenza and parainfluenza viruses) pose a significant burden on families and healthcare systems worldwide. There are an estimated 47,000 cases of LRTI among Canadian children under 5 each year with LRTI mortality occurring in 2.5 Canadian children age 5 and under per 100,000 (95% UI 2.0–3.0) [41]. A study from British Columbia found that LRTI was the primary diagnosis accounting for 32% of hospitalizations for diseases of the respiratory system in children less than 19 years of age and 76% for infants < 1 year of age [42]. An analysis of the burden of RSV on the healthcare system and families in Alberta found that 13.4% of all infants hospitalized with RSV over two seasons had intensive care unit admission, and average ICU stay for these infants was 6.5 days [43]. Lack of breastfeeding has been identified as a risk factor for morbidity and mortality in children with acute lower-respiratory infections. In a systemic review and meta-analysis, Horta and Victora [44] report breastfeeding reduces the prevalence or incidence of respiratory infections (respiratory, lower respiratory tract infection or pneumonia) by 32% [pooled relative risk: 0.68 (95% confidence interval: 0.60; 0.77)], reduces the risk of hospitalization for respiratory infection by 57% [pooled relative risk: 0.43 (95% confidence interval: 0.33; 0.55)] and mortality from lower respiratory tract infections was also reduced [pooled relative risk: 0.30 (95% confidence interval: 0.16; 0.56)].

At the current EBF rate at 6 months (36.3%), the Ontario health system will spend about US $10.3 annually in treating LRTI for the cohort of 2019 newborns who were not EBF. Increasing EBF rate at six months to rate at hospital discharge after delivery and initiation rates would save about 15,028 cases of LRTI per annum and 29,172 cases per annum respectively (Table 2). This would result in about 966–1,791 fewer hospitalisation and 3,487–6,750 fewer outpatient visits for improving current EBF rates at six months to hospital discharge and initiation rates. Such savings in number of cases and healthcare utilisation would save the Ontario health system about US$4.6 million – US$9.1 million in treatment costs annually (Table 2).

**Table 2:** Savings in disease cases and health system costs from improving breastfeeding rates

	Current rate at 6 months (36.3%)		Rate at discharge (64.9%)		Initiation rate (92.1%)	
	Disease cases	Estimated health systems cost	Disease cases averted	Estimated cost savings	Disease cases averted	Estimated cost savings
**Annual health system costs**						
LRTI	32,856	$10,391,974	15,028	$4,665,780	29,172	$9,103,172
GII	27,883	$48,531,464	12,591	$21,911,854	24,441	$42,540,600
AOM	43,174	$8,723,962	19,495	$3,939,414	37,844	$7,647,098
NEC^a^	77	$4,612,636	37	$2,199,854	67	$4,069,568
Total	103,990	$72,260,036	47,151	$32,716,902	67,107	$63,360,438
**Childhood (14-year) health system costs**						
ALL^b^	7	$1,348,340	3	$592,976	6	$1,172,524
Obesity	3,162	$16,823,608	1,199	$6,380,110	2,661	$14,159,081
Asthma	2,492	$42,842,556	970	$16,681,974	2,111	$36,295,822
	5661	$61,014,504	2172	$23,655,060	4778	$51,627,427

^a^NEC – current rate of any breastfeeding at discharge (32.9%); modelled scenario of any breastfeeding at 6 months (64.6%); modelled scenario of improving rate to initiation rate (92.1%)

^b^ALL – current rate of any breastfeeding at 6 months (64.6%); modelled scenario of improving any breastfeeding rates to rates of any breastfeeding at hospital discharge (73.8%); modelled scenario of improving rate to initiation rate (92.1%)

#### Gastrointestinal infections

Sargeant, Majowicz and Snelgrove observed that acute GII represented a significant health burden in Ontario, with a monthly prevalence of 8.6% [24]. They reported that about 1 and 5 Ontarians will seek care from a healthcare professional for GII symptoms including nausea, diarrhea and vomiting. Horta and Victora [44] report “more intense breastfeeding practices” were associated with 31% reduced risk of diarrhea incidence of [pooled relative risk of 0.69 (95% confidence interval: 0.58; 0.82)] compared to “less intense breastfeeding”. The relationship is stronger for infants aged ≤ 6 months, with a corresponding pooled relative risk 0.37 (95% confidence interval: 0.27; 0.50). Breastfeeding also decreased the risk of hospitalization from diarrhea [pooled relative risk: 0.28 (95% confidence interval: 0.16; 0.50) and diarrhea mortality [pooled relative risk: 0.23 (95% confidence interval: 0.13; 0.42)]. Further, Quesada et al. found that the incidence of GII among formula fed children is 17% points higher than the incidence of disease among EBF children [21].

At the current exclusive breastfeeding rate at 6 months (36.3%), the Ontario health system will spend about US $48.5 million annually in treating GII among the 2019 cohort of newborns who were not exclusively breastfed. Increasing exclusive breastfeeding (EBF) rate at six months to rate at hospital discharge after delivery and initiation rates would save about 12,591–24,441 cases of GII per annum respectively (Table 2). This would result in about 2,719–5,305 fewer hospitalisation and 5,392–10,540 fewer outpatient visits for improving current EBF rates at six months to hospital discharge and initiation rates. Such savings in number of cases and healthcare utilisation would result in about US$21.9 million – US$42.5 million savings in treatment costs (Table 2).

#### Acute otitis media

Acute otitis media (AOM) is one of the most common causes of healthcare visits and antibiotic prescriptions for children [39, 45]. In a study of Canadian families who reported having AOM, most (94%, 151 of 161) visited with health professionals and the average time required for medical examination was 3.1 h in an emergency department and 1.8 h in an outpatient clinic [27]. A meta-analysis examining the relationship between AOM and breastfeeding reported that exclusive breastfeeding during the first 6 months is associated with around a 43% reduction in ever having AOM in the first 2 years of life [46].

At the current exclusive breastfeeding rate at 6 months, the Ontario health system will spend about US $6.7 million annually in treating AOM among the 2019 cohort of newborns who were not exclusively breastfed. Increasing EBF rate at six months to rate at hospital discharge after delivery and initiation rates would save about 19,495–37,844 cases of AOM per annum respectively (Table 2). This would result in about 13,164–30,331 fewer outpatient visits for improving current EBF rates at six months to hospital discharge and initiation rates. Such savings in number of cases and healthcare utilisation would result in about US$3.9 million – US$7.6 million savings in treatment costs (Table 2).

#### Necrotizing enterocolitis

Necrotizing enterocolitis (NEC) is one of the most common causes of morbidity and mortality in NICUs, with mortality rates between 15 and 30% [47, 48]. Compared to mother or donor milk, formula feeding preterm or low birth weight infants increases their rate of growth, but nearly doubles the risk of necrotising enterocolitis, a potentially fatal intestinal disease [typical risk ratio (RR) 1.87, 95% CI 1.23 to 2.85; risk difference (RD) 0.03, 95% CI 0.01 to 0.05] [49]. Based on our estimates, about 1,627 newborns in 2019 would be early preterm (about 14% proportion of the 8.1% preterm rate). Of these, about 1,092 would be formula fed at discharge from NICU based on a 32.9% EBF among NICU newborns. Restricting our analyses to these early preterm newborns (i.e. those with < 32weeks gestational age), we estimated potential savings in annual healthcare expenditure in Ontario. At current EBF rate at NICU discharge, the Ontario health system will spend about US $4.6 million per annum to treat the cases of NEC among 2019 early preterm newborns. Increasing exclusive breastmilk feeding (EBM) rates (i.e., mother’s own milk or donor’s milk) at discharge from NICUs in Ontario to the rates of any breastfeeding at 6 months will save about 37 NEC cases, and 67 cases if rates at NICU discharge were to improve to initiation rates (Table 2). This would result in about US$2.1 million – US$4.0 million savings in annual treatment costs (Table 2).

#### Childhood acute lymphoblastic leukemia (ALL)

Childhood cancer is a leading cause of mortality among children and adolescents in high-income countries. In their meta-analysis of 18 case control studies, Amitay and Keinan-Boker [30] estimated that between 14 and 19% of all childhood leukemia cases may be prevented by breastfeeding for 6 months or more. For childhood ALL, we modeled a different breastfeeding scenario. We estimated that there will be about 7 cases of child ALL among the 2019 cohort of newborns attributable to suboptimal breastfeeding. This would result in about US $1.3 million spent in health treatment and management costs over their childhood (14 years). Increasing any breastfeeding rate at six months to rate at hospital discharge and initiation rates would save about 3–6 cases of childhood acute lymphoblastic leukemia for the cohort of children born in Ontario in 2019 (Table 2). These low numbers are because improving any rates to our modelled scenarios would result in only about 9.2% increase in rate. This would result in about US $0.5 million – US $1.1 million savings in childhood (14 years) treatment costs for the 2019 cohort of newborns (Table 2).

#### Childhood obesity

Rao et al. [33] reporting for Statistics Canada and using nationally representative data, found that about 31% of children aged 1–19 are overweight and 13.1% are obese in Canada. This suggests that obesity is a significant public health issue in Canada with estimates similar to those from other high-income countries [50]. A meta-analysis by Ma et al. [34] which included Canadian data found that EBF for 6 months can reduce obesity risk by about 34%.

Using most recent rate estimate of child obesity, about 18,200 of children born in Ontario in 2019 will become obese and incur increased healthcare costs. Further, about 1,737 of cases will be attributed to not exclusively breastfeeding for 6 months. These obesity cases would incur about US $16.8 million in healthcare costs over their childhood (14 years). Increasing EBF rate at six months to rate at hospital discharge after delivery and initiation rates would save about 1,199–2,661 cases of obesity (Table 2). Using evidence of excess annual per capita healthcare costs attributable to child obesity, this would save about US $6.3 million – US $14.1 million in healthcare costs over 14 years (Table 2).

#### Childhood asthma

In Canada, about 3.8 million lives are affected by asthma for children, asthma is the most common chronic disease with a prevalence of 15–25% [51]. This leads to significant healthcare burden for health systems and households as childhood asthma is leading cause of emergency department visits and hospital admissions [31]. Pooling data from 42 studies in primarily high-income countries including Canada, Xue et al. [37] found that EBF for at least 6 months could lower the risk of asthma by about 30%. Using most recent incidence estimates among the cohort of newborns in Ontario in 2019, there will be about 2,492 cases of attributable asthma among not EBF children. Of these, about 99 will be diagnosed at 0–3 years, 1,069 at 4–7 years, and 428 at 8–12 years or age. This would result in about US $42.8 million in healthcare costs before they turn 19 years of age. Increasing EBF rate at six months to rate at hospital discharge after delivery and initiation rates would save about 970–2,111 cases of childhood asthma (Table 2). Using evidence of annual average healthcare costs attributable to childhood asthma. This would save about US $16.6 million – US $36.2 million in healthcare costs (Table 2).

#### Suboptimal breastfeeding and hospitalizations

Overall, our review found that improving exclusive breastfeeding (EBF) rates at six months could significantly reduce hospitalizations. Specifically, we estimate that increasing EBF rates could prevent between 3685 and 7096 hospitalizations for conditions such as lower respiratory tract infections (LRTI) and gastrointestinal infections (GII). (Reference to Supplementary File 1 for hospitalization data).

#### Outpatient visits and common childhood illnesses

Increased EBF rates could also reduce outpatient visits. The review shows that 22,043–47,621 fewer outpatient visits would occur for conditions like LRTI, GII, and acute otitis media (AOM) annually with higher breastfeeding rates. (Reference to Supplementary File 2 for outpatient visit data).

#### Long-term impacts on chronic conditions

Our analysis indicates that increasing breastfeeding rates could result in a reduction of long-term chronic conditions, including obesity, asthma, and acute lymphoblastic leukemia (ALL). Specifically, we estimate that EBF could prevent 1199–2661 cases of childhood obesity, 970–2111 cases of asthma, and 3–6 cases of ALL per year. (Reference to Supplementary File 3 for chronic condition data).

#### Healthcare cost savings

The healthcare cost burden of suboptimal breastfeeding for infants born in Ontario in 2019 is estimated at US $72 million per year for LRTI, GII, AOM, and NEC, and US $61 million in childhood healthcare costs for ALL, obesity, and asthma. By increasing breastfeeding rates, Ontario could save between US $32–63 million annually and US $23–51 million in childhood healthcare costs. (Reference to Supplementary File 4 for cost analysis).

### Sensitivity analysis

Table 3 presents the results of the sensitivity analyses. Due to very high variation in costs from the OCCI, results were most sensitive to the cost parameters used. This is most evident in the estimated costs for the four childhood illnesses (LRTI, GII, AOM, and NEC). For example, using EBF rate at discharge, there is significant differences. Using minimum value for treatment cost estimates for LRTI, cost savings was about US $1.5 million per annum compared to US$119.6 million per annum if maximum treatment cost was used. For child outcomes estimated using odds ratios (ORs), results were most sensitive to varying disease ORs since we relied on treatment costs from published literature which had narrower confidence intervals where available. For these outcomes, a high OR results in fewer cases and lower cost burdens.

**Table 3:** Results from sensitivity analyses for exclusive breastfeeding at discharge and initiation rates, US $ million, 2019 prices

		Varying treatment costs			Varying disease odds ratio		
		Mean estimate	Low estimate	High estimate	Mean estimate	Low estimate	High estimate
**Lower respiratory infection** ^**a**^	Discharge rate	4.3	1.5	119.6			
	Initiation rate	0.971	0.341	26.9			
**Gastrointestinal infection** ^**a**^	Discharge rate	26.6	20.5	228			
	Initiation rate	5.9	4.6	51.4			
**Acute otitis media** ^**a**^	Discharge rate	3.6	3.2	72.4			
	Initiation rate	0.830	0.739	16.3			
**Necrotizing enterocolitis** ^**a**^	Any BF at 6 months	2.4	1.6	3.5			
	Initiation rate	0.543	0.378	0.789			
**Childhood** ^**b**^**acute lymphoblastic leukemia**	Any BF rate at discharge				0.5	0.8	0.3
	Initiation rate				1.1	1.2	0.8
**Child obesity**	Discharge rate	10.4	8.7	2.3	10.4	12.0	8.8
	initiation rate	2.6	2.2	3.1	2.6	3.4	2.0
**Child asthma**	Discharge rate	26.1	16.2	27.7	26.1	30.3	22.2
	Initiation rate	6.5	4.0	6.9	6.5	8.3	5.1

^a^We relied on incidence rates without confidence intervals

^b^data not available on lower and upper bound estimates of healthcare cost

## Discussion

We have reported a conservative estimate of selected pediatric health system costs of suboptimal support for breastfeeding in Ontario, Canada. Improving EBF rates at six months to the rate at hospital discharge or initiation rates could result in 47,114 − 91,457 less cases of common childhood lower-respiratory, gastrointestinal and ear infections. This would result in about 3685–7096 fewer hospitalisations (for LRTI and GII) and 22,043–47,621 fewer outpatient visits (for LRTI, GII, and AOM). There would be approximately 37–67 less cases of NEC per year, 3–6 less cases of ALL per year, 1,199–2661 less cases of childhood obesity, and 970–2111 less cases of childhood asthma.

We found that suboptimal breastfeeding at 6 months of age for infants born in Ontario in 2019 was costing the Ontario healthcare system about US $72.2 million per annum in treatment costs for four childhood illnesses (LRTI, AOM, GII, and NEC), and US $61.0 million in childhood (14 years) treatment and healthcare costs for childhood ALL, childhood obesity and asthma. Increasing breastfeeding rates to our modelled evidence-based scenarios would save about US $32 million – US $63 million in annual treatment cost and US $23.6 million – US $51.6 million in childhood treatment healthcare costs for children born in Ontario in 2019.

We have included an analysis of outcomes where there have been demonstrated robust relationships between human milk and disease prevention in children. We acknowledge that using the term “prevention” in the context of breastfeeding may be a misnomer. Human milk is the normal, biologically appropriate feeding method for human children. It may be argued that breast milk does not prevent disease, as it is the norm. Rather, other forms of feeding, most notably infant formulas, are contributing to disease burden.

This study has several limitations that may impact the accuracy and applicability of the findings. One key limitation is the alignment of breastfeeding rates with the birth cohort utilized in the analysis. While breastfeeding data for Ontario are current, slight discrepancies may exist between the timeline of data collection and the modeling of the birth cohort. These discrepancies could potentially affect the accuracy of our estimates. To mitigate this, we aligned breastfeeding initiation rates from the latest available cohort with current healthcare cost data, though the exact overlap in time frames may still introduce some uncertainty.

Another limitation pertains to the hospitalization cost data used in this study. Although we employed Ontario-specific healthcare costs for childhood illnesses, these figures may not account for all variations in hospitalization costs across different hospitals or regions within the province. Additionally, the hospitalization costs represent averages that may not fully capture the range of healthcare expenditures for more severe cases or for children with comorbidities. The use of static average cost computations, rather than dynamic adjustments, may lead to under- or overestimations of the true financial impact of preventable diseases.

Our analysis primarily focused on the direct impact of breastfeeding on specific childhood illnesses, such as lower respiratory tract infections and gastrointestinal infections. However, many children may experience multiple comorbidities (e.g., asthma and obesity), which could increase both the frequency and cost of hospital visits. The static model used in this study does not account for the compounding effects of these overlapping conditions, suggesting that our estimates may be conservative. Further research could explore the cost implications of comorbidities and how breastfeeding could prevent or mitigate the burden associated with multiple health issues.

In the absence of comprehensive national data on disease incidence linked to breastfeeding in Canada, we incorporated data from other high-income countries with similar healthcare environments, such as the Spain, UK and the US. While this approach allows for a more complete model of disease prevention through breastfeeding, it introduces potential limitations due to differences in healthcare delivery and access. Building on the methodology of the Renfrew et al. study in the UK, we adopted similar adjustments for regional healthcare differences but acknowledge that these variations may affect the applicability of disease incidence rates across jurisdictions.

Our study utilized static models for cost savings estimations, which assume that healthcare costs and disease incidences remain constant over time. This approach simplifies the analysis but may not reflect real-world fluctuations in healthcare costs, disease outbreaks, or changes in healthcare policy. Future studies could benefit from dynamic modeling approaches to better capture the variability in healthcare costs and disease incidence over time. The Renfrew et al. study in the UK provided a foundational framework for our approach, particularly in modeling cost savings from improved breastfeeding rates and utilizing disease incidence data from high-income countries with comparable healthcare systems. Similar to Renfrew et al., we adjusted disease incidence rates to reflect Canadian breastfeeding rates and healthcare costs while recognizing the limitations introduced by healthcare system variations across jurisdictions. It is also noteworthy that Renfrew et al. employed static models for cost estimations, a choice we made for consistency, although dynamic models could yield a more nuanced understanding of cost impacts over time.

We recognize that breastfeeding rates fluctuate over time and that exclusive, partial, and non-breastfeeding rates change as infants grow. In future research, more granular data on breastfeeding practices, including time-varying breastfeeding rates, could be incorporated into the model to reflect these subtleties. This would allow for a more nuanced understanding of how breastfeeding duration and intensity impact pediatric health outcomes.

Additionally, this study does not capture healthcare system costs associated with other diseases linked to a lack of human milk, such as maternal cancers, diabetes, sudden infant death syndrome (SIDS), COVID-19, neonatal abstinence syndrome, maternal obesity, and cognitive deficits [52–67]. Bartick and colleagues have noted that a significant portion (79%) of the medical costs associated with suboptimal breastfeeding are related to maternal health impacts, suggesting that pediatric healthcare costs reported here may be comparatively modest [68]. We did not include the cost of premature deaths for diseases with fatal outcomes, which Bartick and colleagues report as the largest cost of disease [68].

Moreover, we did not account for the costs associated with caregiver time required due to higher rates of childhood illness [69], nor did we calculate the environmental costs of non-human milk products [70]. Capturing the social-emotional costs—including the attachment, bonding, and soothing benefits lost for infants and toddlers weaned early—remains challenging. Additionally, emotional and spiritual costs related to the grief and loss experienced by mothers over breastfeeding relationships that were never established or dissolved prematurely are also difficult to quantify [71].

While the overall cost savings from increased breastfeeding rates are modest in the context of Ontario’s healthcare budget, their significance lies in reducing the operational strain on healthcare services. The financial savings are only one aspect; the real benefit may come from alleviating the burden on scarce healthcare resources and provider time. Emergency department (ED) visits for preventable childhood illnesses like respiratory and gastrointestinal infections consume not only money but also vital resources such as hospital beds, healthcare workers’ time, and emergency care capacity. Increased breastfeeding rates could help reduce pediatric ED visits, which, in turn, would relieve overburdened healthcare providers and hospital systems already facing shortages and burnout. For example, a recent Ontario study found that pediatric ED visits increased by 28.2% from 2008 to 2018 [72], with respiratory diseases accounting for 1 in 3 visits in 2018. Every preventable ED visit avoided by increased breastfeeding represents not just financial savings, but also a reduction in healthcare provider workload and a freeing up of time and hospital resources to treat more critical cases.

Increased breastfeeding rates can lead to significant cost and resource savings for Ontario’s healthcare system and beyond. However, suboptimal breastfeeding should not be seen as a personal failure of mothers or infants; systemic barriers often hinder effective breastfeeding support. While advocates in Canada have successfully raised awareness about breastfeeding benefits, rates remain sub-optimal and require ongoing public health funding and priority. Well-organized and well-funded competitors to breastfeeding advocacy continue to pose challenges. Furthermore, although Canada is a signatory to the International Code of Marketing of Breast-milk Substitutes, enforcement remains lacking, impacting breastfeeding promotion efforts​.

Hospital practices in Canada significantly influence the establishment and maintenance of breastfeeding relationships [73]. The drop in exclusive breastfeeding from birth to discharge may stem from a widespread belief among Ontario healthcare providers that infant formula is a low-risk substitute for breastfeeding [74]. Maternal and child health education should be based on current science regarding human milk and best practices. Efforts to expand Baby-Friendly Hospital Initiative (BFHI) accreditation in Ontario have been limited; in 2021, only 7 of the 93 birthing hospitals, including 51 with NICUs, were BFHI accredited [75]. The costs of accreditation can be prohibitive in a market-driven healthcare system focused on short-term budgeting rather than long-term savings and health outcomes. Our study may offer information for advocates to make a compelling case for evidence-based investments in breastfeeding education and support, reflecting the desires of the majority of birthing individuals in Ontario, leading to improved health outcomes and cost savings.

Other systemic interventions could include expanding midwifery services and implementing mother-centered care models, alongside anti-racism efforts and decolonizing the healthcare system to ensure Indigenous and racialized women have access to culturally relevant, safe prenatal and postnatal care. Community-level interventions should follow evidence-based practices from the BFHI, promoting skin-to-skin contact within the hour after birth and enhancing access to peer support and community breastfeeding resources. For families with infants requiring NICU services, priority should be given to supporting mothers in providing their milk, facilitating skin-to-skin contact, and establishing lasting breastfeeding relationships, along with increased access to donor milk when necessary.

## Conclusion

This study demonstrates that improving exclusive breastfeeding rates in Ontario could significantly reduce the incidence of common pediatric illnesses and alleviate the associated healthcare costs and resource burdens. Our conservative cost analysis highlights the potential for substantial savings in hospitalizations and outpatient visits for conditions such as lower respiratory tract infections, gastrointestinal infections, and acute otitis media. Although breastfeeding initiation rates in Ontario are high, there is a notable decline in exclusivity and duration over time, suggesting an opportunity to strengthen support systems for families. These findings underscore the importance of breastfeeding as a public health strategy that can contribute to improved health outcomes and cost savings for the Ontario healthcare system.

## Supplementary Information

Additional file 1. Search strategy for reviews A, B, and C. Summary of the search strategy used for the reviews.

Additional file 2. Results of review A. The results of Review A.

Additional file 3. Results of review B. The results of Review B.

Additional file 4. Results of review C. The results of Review C.

## Data Availability

The datasets used and/or analysed during the current study are publicly available and sources can be provided by the corresponding authors on reasonable request.

## Abbreviations

AOM: Acute otitis media
BFHI: Baby–friendly health initiative
BFION: Baby–Friendly Initiative Ontario
EBF: Exclusively breastfed
FF: Formula fed
GII: Gastrointestinal infection
LRTI: Lower respiratory tract infection
NEC: Necrotizing enterocolitis
NICU: Neonatal intensive care unit
OCCI: Ontario Case Costing Initiative
RD: Risk difference
RR: Risk ratio
RSV: Respiratory syncytial virus
SIDS: Sudden infant death syndrome
ALL: Acute lymphoblastic leukemia
