# Supplementary material for: The costs of suboptimal breastfeeding in Ontario, Canada, and potential healthcare resource impacts from improving rates: a pediatric health system costing analysis
Source: Int Breastfeed J. 2025 Feb 22;20:9. doi: 10.1186/s13006-025-00702-y (PMC11846301; doi:10.1186/s13006-025-00702-y)
Supplement: Supplementary file 1 — Additional file 1. Search strategy for reviews A, B, and C. Summary of the search strategy used for the reviews. [file 13006_2025_702_MOESM1_ESM.docx]

Supplemental File 1: Search Strategy for Reviews A, B, and C

| Review, Objective | Databases Searched | Search terms (In Title) | Number of Papers Found | Exclusion/Inclusion Criteria and Process of elimination | Number of Articles Used |
| --- | --- | --- | --- | --- | --- |
| Review A  Review of existing review studies examining infant feeding and relationships to health/disease outcomes in high income/medium income countries  Purpose – to use review studies to inform decision making around outcomes to analyze using the rationale described by the Renfrew et al 2012 study | We searched:    Embase -  Cochrane  Database of Systematic Reviews  PubMed -  Google Scholar - | “Breastfeeding” OR “breast fed” OR “bottle feeding” OR “breastfeed” OR “ “bottle feeding” OR “bottle fed” OR “infant feeding” OR “human milk” OR “formula milk” OR “formula feed” OR “human lactation” OR “breast pump” OR “milk express” OR “weaning” AND  “Health outcomes” OR “Developmental outcomes” OR “cognitive outcomes” OR “developmental outcomes” OR “risk factors” OR “causality” OR “mortality” OR “morbidity” OR “health status” OR “women’s health” OR “chronic disease” OR “disease susceptibility” OR “benefit” OR “protective” OR “harmful” OR “cancer” OR “neoplasms” OR “infection” OR “cholesterol” OR “cardiovascular disease” OR “diabetes” OR “intelligence” OR “blood pressure” OR “overweight” OR “obesity” OR “digestive system” OR “allergies” OR “immune system diseases” OR “osteoporosis” OR “dental caries” OR “depression” OR “attention deficit disorder and hyperactivity” OR “developmental disability” OR “attachment” OR “birth intervals” OR “Sudden Infant Death” OR “vaccination” “asthma” OR “Mental health” OR “Menstruation” AND  “review” OR “systematic review” OR “meta-analysis” | Embase: 1899  Cochrane Database of Systematic Reviews: 101  PubMed: 2746  Google Scholar: 738 | Initial review of title/abstract, and screened using inclusions:   - Publication Date: 2012-2023 - Design: Described as a “systematic review” or a “review” and/or “meta-analysis” - Participants: Reviews that include studies with participants from developed/high income countries or transitioning/emerging economies/middle income countries - Exposure: Some or all participants breastfeeding and/or feeding with breast milk (includes hand expression and pumped breast milk fed through means other than the breast) - Outcomes: some form of health and/or cognitive outcomes in relation to breastfeeding/human breast milk   Duplicates removed  Closer reading of articles:   - Only systematic reviews and/or meta-analyses were included in the final - Reviews with Canadian participants/reviews were highlighted for Review B | Embase - 8  Cochrane Database of Systematic  Reviews - 4  PubMed: 153  Google Scholar: 27 |
| Review B  A review of Canadian studies examining health outcomes related to infant feeding  Purpose – to use studies to inform decision making around outcomes to analyze using the rationale described by the Renfrew et al 2012 study | We searched:  CINAHL  PubMed  Google  Scholar | “Breastfeeding” OR “breast fed” OR “bottle feeding” OR “breastfeed” OR “ “bottle feeding” OR “bottle fed” OR “infant feeding” OR “human milk” OR “formula milk” OR “formula feed” OR “human lactation” OR “breast pump” OR “milk express” OR “weaning” AND  “Health outcomes” OR “Developmental outcomes” OR “cognitive outcomes” OR “developmental outcomes” OR “risk factors” OR “causality” OR “mortality” OR “morbidity” OR “health status” OR “women’s health” OR “chronic disease” OR “disease susceptibility” OR “benefit” OR “protective” OR “harmful” OR “cancer” OR “neoplasms” OR “infection” OR “cholesterol” OR “cardiovascular disease” OR “diabetes” OR “intelligence” OR “blood pressure” OR “overweight” OR “obesity” OR “digestive system” OR “allergies” OR “immune system diseases” OR “osteoporosis” OR “dental caries” OR “depression” OR “attention deficit disorder and hyperactivity” OR “developmental disability” OR “attachment” OR “birth intervals” OR “Sudden Infant Death” OR “vaccination” “asthma” OR “Mental health” OR “Menstruation” OR “controlled” OR “experimental” OR “epidemiological” OR “cross-sectional” ; OR “longitudinal” OR “single-group” OR “case control” OR “cohort” AND  “Canadian Studies” OR “Canada” OR “Ontario” OR “Canadians” OR “Canadian” OR “in Canada”  Additional Google Scholar search for specific Canadian study names: “Alberta Case-Control Study on Endometrial Cancer” OR “CHILD Study” OR “Canadian healthy infant longitudinal development” OR “Targetkids” OR “Ontario Health Study” OR “ Nunavut Inuit child health survey” AND “breastfeeding” | CINAHL- 814  PubMed- 2135  Google- 3175  Scholar | Initial review of title/abstract, and screened using inclusions:   - Design: Described as a controlled, experimental or epidemiological (observational or analytic) observational/ descriptive single‐group cross‐sectional or longitudinal studies that include some level of analysis of a relationship between infant feeding and health outcome(s) - Participants: In Canada - Intervention/Exposure: Some or all participants breastfeeding and/or feeding with breast milk (includes hand expression and pumped breast milk fed through means other than the breast (cup, bottle, lactation device) - Outcomes: some form of health and cognitive outcomes in relation to breastfeeding/human breast milk - Any date to 2023 - Exclusions: case reports, case series, and papers that do not report a study   Duplicates removed  Closer reading of the articles: Only research studies conducted with participants living in Canada were included | CINAHL- 13  PubMed- 162  Google- 46  Scholar  Plus the conclusions from Renfrew et al 2012 review of reviews |
| Review C: The cost-of-illness associated with ‘not-breastfeeding’: a systematic examination of evidence reported in developed/transitional countries - high income/medium income  Purpose was to use studies to inform the cost analysis methodology | PubMed EconLit | “Breastfeeding” OR “Human Milk” OR “breast milk” OR “infant feeding” OR “human lactation” OR bottle feeding” OR human milk” OR “milk expression” AND  “cost analysis” OR “cost effectiveness”  OR “cost benefit” OR “cost of illness” OR “Costs and Cost Analysis” OR “Economic Models” OR “economics” | PubMed – 340  EconLit – 74 | Initial review of title/abstract, and screened using inclusions/exclusions:   - Publication Date 2012-2020 - Design: Cost of illness analyses - Participants: Any participants from high income countries or transitioning/emerging economies/middle income countries - excluded only if review indicates they only included studies from low income/developing countries only - Intervention/Exposure: Some or all participants breastfeeding and/or feeding with breast milk (includes hand expression and pumped breast milk fed through means other than the breast (cup, bottle, lactation device) - Analysis of healthcare resource implications of treating conditions that could have been prevented by breastfeeding | PubMed - 31  EconLit – 0  Plus the additional (pre-2012)cost analysis articles found by Renfrew et al 2012 - 11 |
